# Sperm protein profile and their correlation with frozen semen quality of indigenous Indonesian buffalo bulls

**DOI:** 10.5455/javar.2024.k836

**Published:** 2024-12-27

**Authors:** Syahruddin Said, Tulus Maulana, Hikmayani Iskandar, Ekayanti Mulyawati Kaiin, Isyana Khaerunnisa, Widya Pintaka Bayu Putra, Fuad Hasan, Raden Iis Arifiantini

**Affiliations:** 1 Research Center for Applied Zoology, National Research and Innovation Agency, Bogor, Indonesia; 2 Research Center for Animal Husbandry, National Research and Innovation Agency, Bogor, Indonesia; 3 Department of Animal Science, Faculty of Agriculture, Universitas Sumatera Utara, Medan, Indonesia; 4 Division of Veterinary Reproduction and Obstetrics, School of Veterinary Medicine and Biomedical Sciences, IPB University, Bogor, Indonesia

**Keywords:** Buffalo bulls, fertilization ability, sperm protein, sperm quality

## Abstract

**Objective:**

The study aimed to assess sperm motility characteristics, kinematic parameters, and sperm protein molecular weight (MW) in Indonesian buffalo to predict fertility.

**Materials and Methods:**

Frozen semen from Silangit (4 bulls), Murrah (4 bulls), and Toraya (2 bulls)—aged 7–10 years, was analyzed. Sperm motility was assessed using Computer Assisted Semen Analysis, viability and abnormality were evaluated using eosin-nigrosin staining, plasma membrane integrity was evaluated using the hypoosmotic swelling test, acrosomal status was evaluated using lectin peanut agglutinin, protamine deficiency was evaluated using chromomycin A3, and deoxyribonucleic acid (DNA) integrity was evaluated using Halomax. Protein concentration was determined using the bicinchoninic acid method and characterized with sodium dodecyl sulfate-polyacrylamide gel electrophoresis.

**Results:**

The study revealed breed-specific variations in semen quality. Silangit buffaloes exhibited lower DNA integrity, while Murrah buffaloes showed elevated motility and membrane integrity. Toraya buffaloes displayed higher normal morphology and protamine status, though they had lower viability. Notable differences in protein expression included the presence of SPAG9 and the absence of IZUMO1 in Toraya buffaloes. Protein MW analysis further showed correlations with sperm characteristics. In Murrah buffaloes, proteins within the 130–125 kilodalton (kDa) range were negatively correlated with acrosome integrity, whereas in Toraya buffaloes, proteins within the 55–50 kDa range were negatively correlated with sperm abnormalities. Silangit buffaloes showed a positive correlation between proteins at 32 kDa and sperm abnormalities.

**Conclusion:**

Analyzing protein MW through SDS-PAGE provides a promising approach for assessing semen quality in indigenous Indonesian buffalo bulls. Although the semen quality of the buffaloes in this study was variable, all bulls met the established Indonesian standards for semen quality and exhibited adequate fertilization potential. These results provide valuable insights into the reproductive biology of Indonesian buffalo bulls and form the basis for predicting fertility capacity through a comprehensive analysis of sperm characteristics and molecular profiles of sperm proteins.

## Introduction

**Figure 1.**
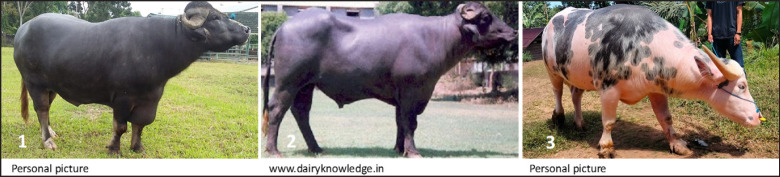
Local Indonesian buffaloes. 1: Silangit buffalo; 2: Murrah buffalo; 3: Toraya buffalo.

Indonesia is a country with outstanding biodiversity and a wide range of genetic resources for livestock, including indigenous, local, and introduced animals. Buffalo is a livestock species that is still widely distributed and developed in Southeast Asia, especially in Indonesia [1]. Several well-developed local buffalo breeds exist in Indonesia, including Silangit, Murrah, and Toraya buffalo (Fig. 1).

Buffalo meat has significant human health benefits, including lower cholesterol content than beef. Buffalo milk has higher protein content, fewer somatic cells, and less cholesterol [2], higher milk quality (higher solids content) and meat (lower intramuscular fat content), higher resistance to parasitic infections, good feed conversion, and low maintenance requirements that are also profitable for farmers. Despite these advantages, buffaloes have lower reproductive performance than other animals worldwide. They are prone to various reproductive disorders, including delayed puberty, inadequate expression of estrus, prolonged ovarian inactivity after birth, and significantly reduced conception rates, particularly when artificial breeding methods are used.

Semen quality assessment provides valuable information about the reproductive potential and fertility of bulls, which is essential for successful breeding and genetic improvement programs. By evaluating semen quality parameters such as sperm concentration, motility, morphology, viability, and deoxyribonucleic acid (DNA) integrity, breeders can make informed decisions about selecting better sires for artificial insemination (AIC) and natural breeding. The fluorescent staining technology uses fluorescent dyes that bind either indirectly or directly to a portion of the sperm function or structure, such as the membrane, mitochondria, acrosome, or DNA. Various fluorescent staining techniques have examined sperm viability, acrosome state, and mitochondrial activity. These parameters are considered useful for *in vitro* testing and predicting fertility in the field [3]. The advent of computer-assisted semen analysis (CASA) has provided a means to obtain objective data on various aspects of sperm quality, including progression and kinetic parameters. CASA provides valuable insights into several parameters, including progressive motility (pMOT), average path velocity (VAP), curvilinear velocity (VCL), lateral head displacement (ALH), straight-line velocity (VSL), and beat-cross frequency (BCF) [4].

Conventional semen parameters are currently considered insufficient for predicting the fertility of a bull. Consequently, genetic markers are needed for a more accurate prediction of fertility rates and contribute to the selection of bulls. The use of genes and proteins in sperm and seminal plasma as molecular markers in combination with semen quality evaluation has been widely reported and is thought to be more effective.

Comprehensive data on the reproductive performance of buffalo bulls is still limited. Therefore, this study aims to evaluate the characteristics of semen quality, CASA motility, kinetic parameters, and fluorescein staining results of cryopreserved bull semen and the profile of the MW of sperm proteins in Indonesian buffalo bulls to predict the fertility capacity of bulls. These results provide valuable insights into the reproductive biology of Indonesian buffalo bulls and form the basis for fertility prediction through a comprehensive analysis of semen characteristics and sperm protein molecular profiles.

## Materials and Methods

### Ethical approval

This study was approved by the Animal Ethics Committee of the National Research and Innovation Agency (BRIN) under certificate number 093/KE.02/SK/05/2023, regarding the use of animal models and experimental design. It included three Indonesian buffalo breeds: Silangit (4 bulls), Murrah (4 bulls), and Toraya (2 bulls), aged 7–10 years. These buffaloes were maintained by current ethical guidelines for animal care at the Artificial Insemination Centre (AIC).

### Study period and location

This study was conducted from May to November 2023. Frozen semen from Murrah buffaloes was obtained from the National AIC in Lembang Bandung, West Java; Silangit buffaloes from the Regional AIC in Medan, North Sumatera; and Toraya buffaloes from the Regional AIC in Pucak, South Sulawesi. Analyses of sperm quality characteristics, CASA motility, kinetic parameters, immunofluorescence of cryopreserved semen, and the molecular weight (MW) profiles of sperm proteins were carried out at the Research Centre for Applied Zoology, BRIN.

### Sperm characteristics of frozen semen

The stained slides were evaluated using a light microscope at 400 × magnification, and at least 400 sperm cells were analyzed in each sample. Values for viability, abnormalities, and membrane integrity are expressed as a percentage (%).

The acrosomal status of semen samples was evaluated by using lectin peanut agglutinin (FITC-PNA, Sigma St. Luis MO) fluorescence stain combined with propidium iodide. The protamine status of each sperm cell was determined using the chromomycin A3 (CMA3, Sigma St. Luis MO) technique. To evaluate the DNA integrity of frozen-thawed sperm was evaluated by using the Halomax (Halotech DNA S.L. Spain) staining technique [5].

### Computer-assisted semen analysis

Sperm motility was assessed by CASA at a temperature of 38°C using the Sperm Vision Program (Minitub, Tiefenbach, Germany). Parameters measured included sperm kinematics progressive motility (Pmot%), VCL (velocity curved line μm/second), velocity straight line (VSL μm/second), velocity average path (VAP μm/second), amplitude of lateral head displacement (ALH μm), beat cross frequency (BCF Hz), straightness (STR%), velocity straight line (VSL μm/second), velocity of the average path (VAP μm/second), linearity (LIN%), and wobble (WOB%).

### Determination of frozen semen protein

The frozen semen was thawed in a water bath at 37°C for 30 seconds, then washed three times with phosphate-buffer saline and centrifuged at 1800 rpm. The sperm pellet was subsequently extracted using PRO-PREP^TM ^protein extraction solution (iNtRON Biotechnologi, Korea) according to the manufacturer’s instructions. The total soluble protein concentration of the sample was measured by the bicinchoninic acid (BCA) method for colorimetric detection and quantitation of total protein with Pierce™️ BCA Protein Assay Kit 23225 (Thermo Scientific™, USA) before sodium dodecyl sulfate-polyacrylamide gel electrophoresis (SDS-PAGE) analysis. SDS-PAGE analysis was performed to determine the protein profile based on MW, which was visualized as bands on the gel. Protein separation was performed using a Precast Gel SDS-PAGE, M01210 (ExpressPlus, Genscript Biotech Corp., Hong Kong), with a 12% polyacrylamide gel containing sodium dodecyl sulfate (SDS). The separation was carried out at 140 V and 75 mA for 55 min. The gel was then stained with coomassie brilliant blue (CBB). The marker used was the Broad Multi-Color Pre-Stained Protein Standard M00624 (Genscript Biotech Corp., Hong Kong), with a MW range of approximately 5–270 kilodalton (kDa). The differential intensity of each protein band was then quantified using ratio analysis with ImageJ software.

### Data analysis

The research data were analyzed using Minitab Statistical Software version 18.1 (Minitab for Windows, Minitab, Inc., USA). A normality test was performed on the data using the Shapiro-Wilk test, followed by the Levene test for homogeneity. As the data were normally distributed and exhibited homogeneity of variance, analysis proceeded with one-way analysis of variance. Tukey’s post-hoc test was conducted to assess differences between the variables tested. Spearman’s correlation analysis was used to evaluate the relationship between sperm protein MW and sperm quality.

## Results and Discussion

### Sperm characteristics of frozen semen Indonesia buffalo bulls

The percentage characteristics of sperm from Buffalo bulls’ frozen semen are presented in Table 1. Overall, frozen semen from Murrah, Silangit, and Toraya buffalo bulls met the Indonesian national standards for frozen buffalo semen, which require post-thawing motility to exceed 40% (SNI 4869–2:2021). As shown in Table 1, buffalo semen exhibits a range of characteristics. The DNA integrity of Silangit buffalo sperm was significantly lower (*p* < 0.05) compared to the other buffalo breeds. Sperm motility and plasma membrane integrity were significantly higher in Murrah buffaloes (*p* < 0.05). The percentage of normal morphology and protamine status was significantly greater in Toraya buffaloes (*p* < 0.05), while sperm viability was significantly lower (*p* < 0.05) compared to the other breeds.

The results showed that Murrah buffaloes exhibited significantly higher sperm motility, plasma membrane integrity, and acrosomal integrity. In contrast, the DNA integrity of Silangit buffalo sperm was significantly lower (*p < *0.05) compared to other buffalo breeds. In comparison, Toraya buffalo showed significantly higher proportions of normal sperm morphology and protamine status (*p < *0.05). However, their sperm viability was significantly lower compared to the other buffalo breeds (*p < *0.05) (Table 1).

Sperm motility has been positively correlated with bull fertility [6]. Among all semen quality parameters, sperm motility is considered a key predictor of male fertility potential [7]. High levels of motility are associated with enhanced mitochondrial capacity to generate adenosine triphosphate (ATP), which is essential in maintaining sperm flagella movement [8]. Sperm motility relies on the availability of sufficient energy, primarily derived from ATP, which is generated along the axoneme-microtubule pathway through the hydrolysis of dynein ATPase [9]. It is important to note that the energy required for sperm movement results from the interplay between glycolysis and oxidative phosphorylation mechanisms, which together contribute to ATP production within the microtubules [10].

**Table 1.** Sperm characteristics of frozen semen Indonesian buffalo bulls.

Parameters	Buffalo bulls (Mean ± SD)		
	Silangit	Murrah	Toraya
Motility (%)	60.57 ± 5.96^a^	66.46 ± 2.7^b^	62.63 ± 2.33^a^
Viability (%)	66.85 ± 2.82^a^	69.24 ± 4.52^a^	59.49 ± 3.82^c^
Abnormality (%)	10.16 ± 1.19^a^	6.88 ± 0.95^b^	4.55 ± 0.69^c^
Plasma membrane Integrity (%)	63.11 ± 2.91^a^	66.67 ± 3.74^b^	61.26 ± 1.44^a^
Acrosomal integrity (%)	97.71 ± 1.09^a^	95.46 ± 0.68^b^	97.61 ± 0.64^a^
Protamine status (%)	95.54 ± 2.11^a^	94.93 ± 1.23^a^	97.97 ± 1.37^b^
DNA integrity (%)	89.74 ± 3.54^a^	96.67 ± 3.62^b^	96.09 ± 2.12^b^

Means in the same row with different superscripts differ significantly (*p < *0.05).

The viability of sperm is one of the determining parameters for the quality of sperm. It is determined by the number of surviving sperm cells, which is assessed using the eosin-nigrosine staining procedure. Frozen semen with a sperm viability percentage ranging from 64% to 80% is considered suitable for AI. In this study, the sperm viability of Silangit buffaloes (66.85 ± 2.85), Murrah buffaloes (69.24 ± 4.52), and Toraya buffaloes (59.49 ± 3.82) indicated good quality sperm for AI. These results were previously reported by Ansari et al. [11], using frozen semen from Nili-Ravi buffaloes.

In this study, analysis of abnormal sperm morphology showed significantly higher value in Silangit buffaloes (10.16 ± 1.19) compared to Murrah buffaloes (6.88 ± 0.95) and Toraya buffaloes (4.55 ± 0.69) (Table 1). The sperm abnormality rate is 31.86% in swamp buffaloes. Stress caused by elevated temperatures is particularly detrimental to sperm quality. Furthermore, inadequate nutrition and the stress resulting from a combination of environmental factors and pathogens can negatively impact spermatogenesis and spermiation [12]. Malfunctions during spermatogenesis or spermiogenesis, along with genetic factors, diseases, and inappropriate environmental conditions, contribute to primary sperm abnormalities. Abnormalities in any part or organelle of the sperm can compromise sperm motility [13].

The integrity of the plasma membrane is a critical determinant of sperm functionality, as it directly influences metabolic processes and is closely associated with sperm motility and viability [14]. The plasma membrane plays a pivotal role in maintaining osmotic balance, facilitating the transport of essential substances, and preserving the structural integrity of both the mitochondrial membrane and acrosome. Disruption of the plasma membrane compromises sperm motility, viability, and acrosomal integrity. In the present study, plasma membrane integrity was significantly higher in Murrah buffaloes (66.67 ± 3.74) compared to Silangit buffaloes (63.11 ± 2.91) and Toraya buffaloes (61.26 ± 1.44). Sperm motility is generally expected to be in accordance with or exceed the integrity of the plasma membrane. The sperm motility observed in the buffalo bulls in this study ranged between 60% and 66%. Membrane damage leads to a loss of motility, accelerated metabolic disturbances, morphological changes, disintegration of the acrosomal cap, and the release of intracellular components.

Fluorescent labeling of the plasma membrane and organelles of sperm has been widely employed to assess various sperm quality parameters, including viability, acrosome integrity, and mitochondrial function. In this study, acrosome integrity (Table 1) showed a significant difference between Silangit buffalo (97.71 ± 1.09), Murrah buffalo (95.46 ± 0.68), and Toraya buffalo (97.61 ± 0.64). Acrosome integrity is a key determinant of fertility, as it plays a critical role in sperm function during fertilization.

For successful fertilization through AI, sperm must retain intact acrosomes upon insemination into the female reproductive tract. Upon reaching the fertilization site, sperm must undergo an immediate acrosomal reaction (AR) [15]. The acrosome mediates the AR, a vital process for the fusion between sperm and oocyte. During this reaction, the sperm’s plasma membrane fuses with the outer acrosomal membrane, leading to the exocytosis of acrosomal contents. This fusion activates acrosomal enzymes, enabling sperm to penetrate the zona pellucida. The integrity of the acrosome is essential for successful fertilization, as premature AR before oocyte contact results in sperm incapacity to fertilize the oocyte.

Protamine deficiency was assessed using fluorescent CMA3 staining, as shown in Figure 2.3 and Table 1. The results showed no significant difference in protamine deficiency between the Silangit buffalo (95.54 ± 2.19) and Murrah buffalo (94.93 ± 1.23), although both exhibited significantly lower values compared to the Toraya buffalo (97.97 ± 1.37), Protamine deficiency was found to be below 6% in both buffalo breeds. This finding is slightly higher than that reported in Bali bulls (protamine deficiency < 5%) by Hasbi et al. [16] and in Simmental bulls (protamine deficiency < 4%) by Baharun et al. [17]. However, the observed deficiency was lower than the 23.05% protamine deficiency reported by Carreira et al. [18]. CMA3, a guanine-cytosine-specific fluorochrome, competes with protamine binding sites on DNA, causing chromomycin to bind to DNA, which leads to fluorescence in sperm with low protamine levels. Disruption of protamine binding can result in significant DNA instability, leading to elevated levels of DNA damage [19] and morphological abnormalities in sperm heads.

In this study, DNA integrity assessed using the Halomax method was significantly lower (*p < *0.01) in Silangit buffalo bulls (89.74 ± 3.54) than in Murrah (96.67 ± 3.62) and Toraya buffalo bulls (96.09 ± 2.12) (Table 1, Fig. 2.2). DNA damage levels of 3%–8% have been reported in Simmental bulls [17], while it is less than 5% in Bali cattle [5]. A reduction in male fertility when DNA damage exceeded 10%. Previous studies have reported that DNA damage below 15% was considered normal, whereas levels between 15% and 25% were associated with reduced fertility [20]. DNA damage of more than 25% was classified as a sign of infertility [20]. The results of this study with buffalo bulls showed that the DNA fragmentation rates in buffalo bulls were within the established acceptable range, suggesting that sperm quality remained intact despite the freezing process. Oxidative stress is recognized as a major contributor to sperm DNA damage, leading to compromised sperm membrane integrity and reduced motility [21]. Furthermore, elevated ambient temperatures can increase testicular heat, exacerbating DNA damage in sperm.

**Figure 2.**
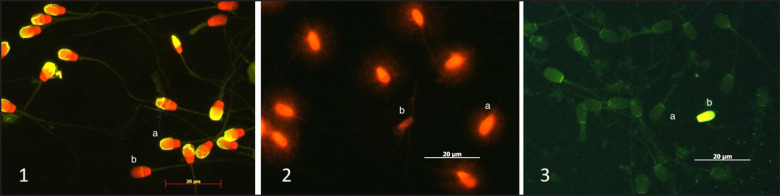
Magnification 630x, microscope fluorescence, Imager Z7, Carl Zeiss, Germany. 1: Acrosomal status; 1a: Intact acrosome (yellow fluorescence in the acrosome); 1b:non-intact acrosome (red fluorescence no acrosome); 2: DNA integrity; 2a: Normal DNA (intact fluorescence); 2b: Fragmented DNA (shrink-dimmer fluorescence); 3: Protamine status; 3a: Normal/complete protamine (dark/dull green fluorescence); 3b: Protamine deficiency (yellow fluorescence).

### Computer-assisted semen analysis

Table 2 showed that the CASA movement patterns, including pMot, (DCL), DSL, and VCL, were not significantly different between the buffaloes (*p > *0.05). However, the movement parameters of DAP, VAP, and ALH were significantly lower (*p < *0.05) in Silangit buffaloes compared to other buffaloes. Conversely, Murrah buffaloes showed significantly higher values (*p* < 0.05) for VCL, LIN, and WOB, while Toraya buffaloes exhibited significantly lower (*p* < 0.05) LIN values. These results indicate significant differences in motility and kinetic characteristics among Silangit, Murrah, and Toraya buffaloes.

Sperm motility is a critical indicator of sperm viability and structural integrity, playing a pivotal role in determining fertilization potential. The CASA provides precise and accurate data on various characteristics of sperm motility. In recent decades, the role of sperm motility in fertilization has garnered considerable attention, with motility now universally acknowledged as a fundamental criterion in evaluating sperm fertilizing ability [22]. Furthermore, motility parameters such as the ALH, VSL, VCL, and LIN have been reported to correlate with fertility in humans [23].

In this study, sperm motility was significantly higher (*p < *0.05) in Murrah buffaloes compared to Silangit and Toraya buffaloes (Table 1). However, there was no difference in pMOT (Table 2). These results showed higher values compared to previous studies, where frozen Murrah buffalo semen had motility and pMOT of 57.41 ± 0.92 and 23.56 ± 0.60, respectively [24]. This difference could be due to differences in the diluent used in freezing the sperm. The diluent in this study was skimmed-egg yolk, whereas tris-egg yolk was used in previous studies.

**Table 2.** Computer-assisted semen analysis movement patterns in the sperm of frozen semen Indonesian buffalo.

Parameters	Buffalo bulls (Mean ± SD)		
	Silangit	Murrah	Toraya
Progressive motility, pMot (%)	53.11 ± 8.01	53.76 ± 6.25	52.52 ± 2.71
Distance average path, DAP (μm/second)	26.46 ± 4.95^a^	31.04 ± 6.25^b^	29.42 ± 3.04^b^
Distance curve length, DCL (μm/second)	41.42 ± 6.88	42.52 ± 6.25	41.78 ± 3.04
Distance straight length, DSL (μm/second)	22.23 ± 4.15	23.19 ± 4.76	20.38 ± 1.89
Velocity average path, VAP (μm/second)	62.34 ± 8.01^a^	71.40 ± 7.76^b^	69.89 ± 3.97^b^
Velocity curve length, VCL (μm/second)	101.21 ± 7.87	108.99 ± 9.04	101.18 ± 4.18
Velocity straight length, VSL (μm/second)	47.72 ± 4.56^a^	57.38 ± 4.42^b^	47.82 ± 3.75^a^
Straightness, STR (%)	73.56 ± 3.41^a^	73.25 ± 5.31^a^	66.87 ± 2.06^b^
Linearity, LIN (%)	50.5 ± 3.36^a^	53.50 ± 3.86^b^	46.81 ± 2.71^c^
Wobble, WOB (%)	67.25 ± 3.39^a^	71.18 ± 2.10^b^	68.19 ± 1.22^a^
Amplitude of lateral head, ALH (μm)	3.93 ± 0.7^a^	4.34 ± 0.74^b^	5.29 ± 0.72^b^
Beat cross frequency, BCF (Hz)	27.08 ± 3.31^a^	28.61 ± 3.82^a^	23.04 ± 1.04^b^

Means in the same row with different superscripts differ significantly (*p < *0.05).

The DCL measures the distance sperm moves per second along a curved path. In this study, sperm showed no difference in DAP, DCL, and DSL between Silangit, Murrah, and Toraya bulls after thawing.

The sperm velocity parameters, including VAP and VSL, exhibited significant differences (*p* < 0.05) between the buffalo bulls after thawing. Specifically, the VAP was significantly lower (*p* < 0.05) in the Silangit bull compared to the other bulls, while the VSL was significantly higher (*p* < 0.05) in the Murrah bull. The evaluation of VAP, VSL, and VCL is crucial for assessing sperm quality, as both VAP and VCL are positively correlated with fertility. Notably, bulls with high fertility typically exhibit significantly higher VAP and VCL values than bulls with medium or low fertility. Sperm movement is considered to possess strong fertility potential when VCL > 70 μm/second, VSL > 70 μm/second, and VAP > 45 μm/second [25]. Based on these criteria, the results of this study suggest that Silangit, Murrah, and Toraya bulls demonstrate robust fertility potential, evidenced by their “rapid” motility, with VCL exceeding 90 μm/second. In terms of sperm kinematics, the STR and LIN parameters were significantly lower (*p* < 0.05) in the Toraya bull compared to the Murrah and Silangit bulls. Additionally, when comparing all three buffalo breeds, the Murrah bull showed significantly higher WOB, the Silangit bull exhibited significantly lower ALH, and the Toraya bull demonstrated significantly lower BCF. Overall, the kinematic analysis of sperm in this study indicates that all buffalo bulls, regardless of breed, exhibit substantial fertility potential. 

### Sperm protein profiles of buffalo bulls assessed by 1D-SDS page

The sperm protein profile based on MW as analyzed by 1D-SDS PAGE is presented in Table 3. A total of 10 protein bands were identified, with MWs of 175 kDa, 130–125 kDa, 95–90 kDa, 85–75 kDa, 65 kDa, 55–50 kDa, 45 kDa, 35 kDa, 32 kDa, and 30–5 kDa. Among these, the protein bands in the ranges of 130–125 kDa, 95–90 kDa, 55–50 kDa, 35 kDa, 32 kDa, and 30–5 kDa were identified as HYOU1, Arylsulfatase-a, ß-protein-N-acetyl-guicosaminidase, glutathione peroxidase 3, sperm acrosome membrane-associated protein 1, BSP A1/A2, BSP-A3, and BSP-30, respectively, in Silangit, Murrah, and Toraya buffaloes. These protein markers are essential for sperm function and are indicative of key physiological processes such as acrosomal reaction, antioxidant defense, and membrane stability.

A correlation between sperm quality and bull fertility has been reported in various species, including cattle [17], goats [26], and horses [27]. However, studies on sperm proteins in buffalo remain relatively limited. Sperm undergoes interactions with a wide range of proteins at different stages of their lifecycle, from spermatogenesis to ejaculation. These interactions are integral to their fertilizing capability [28]. The expression of proteins involved in biological functions such as reproduction, stress response, and metabolic processes significantly influences sperm function. Proteins regulating motility, immunity, capacitation, adhesion, acrosome reaction (AR), and zona pellucida binding play a crucial role in determining fertilization potential [29]. Variations in the expression of the SPAG9 protein among Silangit, Murrah, and Toraya buffaloes may be linked to genetic differences that impact protein regulation, sperm quality, and overall productivity. Toraya buffaloes may harbor unique genetic traits or variations that influence the expression of the SPAG9 protein. Genetic diversity within buffalo populations contributes to variability in protein expression patterns, and the distinct presence of SPAG9 in the acrosomal compartment underscores its role in sperm-ovum interaction. Studies focusing on tissue-specific expression have revealed that SPAG9 transcripts are exclusively localized in the testis, highlighting their relevance to sperm function and fertilization.

**Table 3.** Sperm protein profiles of buffalo bulls assessed by 1D-SDS page.

Proteins	References	MW (kDa)	Buffalo Bulls		
			Silangit	Murrah	Toraya
Sperm-associated antigen 9 (SPAG9)	[29]	175	−	−	+
Hypoxia up-regulated 1 (HYOU1)	[30]	130–125	+	+	+
Arylsulfatase-a	[31]	95–90	+	+	+
Heat shock-protein 70 (HSP70)	[33]	85–75	+	−	−
Heat stock-protein 2 (HSP2)	[32]	65	−	−	+
ß-N-asetil-guicosaminidase	[36]	55–50	+	+	+
Izumo sperm-egg fusion protein 1 (IZUMO1)	[34,35]	45	+	+	−
Glutathione peroxidase 3	[37]	35	+	+	+
Sperm acrosome membrane-associated protein 1 (SPACA1)	[38,39]	32	+	+	+
Binder sperm protein (BSP) A1/A2, BSP-A3 dan BSP-30 (BSP1, BSP3, dan BSP5)	[39]	30–5	+	+	+

A protein with a MW of 130–125 kDa has been identified as the HYOU1 protein in Silangit, Murrah, and Toraya buffaloes. HYOU1 is part of a large multi-complex aggregate associated with cytoprotective mechanisms. The presence of this 130–125 kDa protein suggests a conserved role in response to specific environmental stressors, potentially involving temperature regulation, oxidative stress, or biological processes related to hypoxia. HYOU1 has been found in the plasma membranes of human sperm, highlighting its relevance in cellular protection [30].

The presence of the MW of 95–90 kDa protein in Silangit, Murrah, and Toraya buffaloes indicates uniform expression of the Aylsulfatase-a protein. This protein is involved in the breakdown of sulfur compounds in biological processes involving sulfate groups. Moura et al. [30] reported that Arylsulfatase-a has MWs between 71 and 100 kDa, a protein from the cauda epididymis. Aylsulfatase-a plays a role as a molecular receptor complex in the process of sperm-oocyte recognition, and it plays a role in male fertility [31].

Variations in the expression of heat shock proteins (HSP70 and HSP2) in Silangit, Murrah, and Toraya buffaloes indicated different responses to heat stress. This is related to environmental differences or genetic adaptations between the buffalo species. Heat shock proteins form a group of structurally unrelated proteins that are categorized into the HSP100 (HSPH), HSP90 (HSPC), HSP70 (HSPA), HSP60 (HSPD), and HSP27 (HSPB) families in mammals [32]. Heat shock proteins have been detected on the surface of mouse, rat, bull, boar, and human sperm, and HSP70 family members appear to be common components of the sperm surface [33].

The MW 45 kDa was expressed in both Silangit and Murrah buffaloes and was identified as IZUMO1. This protein plays a crucial role in facilitating the AR, an essential process for sperm-egg fusion. However, the absence of the MW 45 kDa in Toraya buffalo suggests genetic or regulatory differences among buffalo breeds. Further research is needed to better understand the mechanisms underlying IZUMO1 expression and its implications for reproductive biology in buffalo populations.

Protein bands of 26 and 55 kDa were reported in highly fertile bulls. Fukuda et al. [34] reported the expression of IZUMO1 in the testis, epididymis, and liver of Japanese Black cattle. IZUMO1 is involved in the fusion of sperm and egg. The MWs of IZUMO1 in ejaculated boar sperm were found to be 42 kDa and 45 kDa in bulls [35]. The presence of ß-N-acetyl-glucosaminidase protein in all buffaloes indicates a conservative role in metabolic and fertilization processes. Kausar et al. [36] reported β-N-acetyl-glucosaminidase activity in milk samples obtained from uninfected mammary glands of Nilli-Ravi buffaloes, Sahiwal cows, and cross-bred cows to elucidate the enzymatic activity of NAGase and its potential impact for mammary gland health in these specific breeds of NAGase, which is known for its involvement in the degradation of glycosaminoglycan, could play a crucial role in understanding the health status, especially related to infections, in the mammary glands of Nilli-Ravi buffaloes, Sahiwal cows, and cross-bred cows.

Glutathione peroxidase 3 is expressed at MW 35 kDa in Silangit, Murrah, and Toraya buffalo. These proteins play a role in protecting cells from oxidative damage and are associated with the acrosomal membrane of sperm. Glutathione peroxidase 3 specifically protects sperm cells from free radical-induced damage, as highlighted by previous studies [37]. The presence of phospholipid hydroperoxide glutathione in bull sperm serves as a distinctive indicator and unique marker of analyzing semen quality.

SPACA1, detected in Silangit, Murrah, and Toraya buffaloes at a MW of 32 kDa, is another important protein involved in sperm function. According to Rosyada et al. [38], SPACA1 is expressed in the epididymis during sperm maturation and plays a crucial role in maintaining acrosome and sperm fertility. The expression of SPACA1 in these buffalo breeds suggests its involvement in sperm functionality, which is essential for successful fertilization [33].

The BSP protein, with an MW of 5–30 kDa, is present in Silangit, Murrah, and Toraya buffaloes. The presence of BSP has indicated its function and interaction with sperm, is associated with fertility, and contributes to the maintenance of sperm integrity. Some studies on BSPs have been reported in bulls, where BSPs are known to interact with sperm during ejaculation, play a role in capacitation, facilitate the interaction between sperm and oviductal epithelia, and influence the process of fertilization [39]. BSP1 is a low MW glycosylated protein and the most abundant among all BSPs.

### Correlation between sperm protein MW and sperm quality

The analysis results showed a significant correlation (*p < *0.05) between MW of sperm protein and semen quality, as presented in Table 4. Proteins with a MW of 130–125 kDa showed a negative correlation (–0.990) with acrosome integrity in Murrah buffaloes, while those with a MW of 85–75 kDa showed a positive correlation (+0.990). Proteins with a MW of 55–50 kDa showed a negative correlation (–0.984) with sperm abnormalities in Toraya buffaloes. Conversely, a positive correlation (+0.959) was found between the protein with an MW of 32 kDa and the sperm abnormalities in Silangit buffalo.

The present study found a correlation between sperm protein MW and sperm quality in Indonesian buffalo bulls. Proteins with a MW of 130–125 kDa showed a negative correlation (–0.990) with acrosome integrity in Murrah buffalo, while those with a MW of 85–75 kDa showed a positive correlation (+0.990). Proteins with a MW of 55–50 kDa showed a negative correlation (–0.984) with the abnormalities in Toraya buffaloes. Additionally, a positive correlation (+0.959) was found between the protein with a MW of 32 kDa and the sperm abnormalities in Silangit buffaloes. These results suggest that specific proteins play a role in the normal spermatogenesis process. Fu et al. [28] reported that seminal plasma proteins are involved in sperm maturation through the activated protein kinase pathway. Sperm motility and morphology are crucial factors in determining the fertility or fertilization success of bulls. To fertilize the oocyte, sperm must be alive, motile (with progressive movement), have normal morphology, and possess intact DNA [40]. The correlations observed in this study regarding molecular proteins in sperm provide additional insights for selecting superior bull candidates.

**Table 4.** Correlation between MW of sperm protein and sperm quality.

MW (kDa)	Silangit				Murrah				Toraya			
	Motility	Acrosome integrity	Abnormality	Membrane plasma integrity	Motility	Acrosome integrity	Abnormality	Membrane plasma integrity	Motility	Acrosome integrity	Abnormality	Membrane plasma integrity
175	−	−	−	−	−	−	−	−	(+) 0.474	(−) 0.919	(–) 0.634	(–) 0.628
130–125	(+) 0.385	(+) 0.341	(−) 0.791	(−) 0.923	(−)0.577	(−)0.990*	(+)0.843	(−)0.724	(−) 0.324	(+) 0.644	(–) 0.510	(+) 0.817
95–90	(−) 0.608	(+) 0.592	(−) 0.292	(−) 0.036	(−)0.205	(+)0.424	(−)0.061	(−)0.102	(+) 0.629	0	(+) 0.650	(–) 0.451
85–75	(−) 0.517	(−) 0.002	(+) 0.678	(+) 0.378	(−)0.579	(+)0.990*	(−)0.844	(−)0.725				
65	−	−	−	−	−	−	−	−	(+) 0.780	(–) 0.276	(–) 0.497	(–) 0.263
55–50	(−) 0.740	(+) 0.931	(−) 0.404	(−) 0.581	(+)0.675	(+)0.748	(−)0.672	(+)0.718	(+) 0.188	−) 0.249	(−) 0.984*	(+) 0.133
45	(−) 0.198	(+) 0.809	(−) 0.935	(−) 0.827	(+)0.713	(+)0.782	(−)0.906	(+)0.806	(+) 0.614	(+) 0.066	(+) 0.616	(−) 0.380
35	(+) 0.516	(+) 0.003	(−) 0.679	(−) 0.379	(−)0.263	(+)0.600	(−)0.131	(−)0.072	(+) 0.455	(−) 0.879	(−) 0.706	(−) 0.562
32	(+) 0.144	(–) 0.793	(+) 0.959*	(+) 0.871	(+)0.180	(–)0.200	(+)0.128	(+)0.065	(+) 0.630	(–) 0.001	(+) 0.649	(−) 0.452
30	(+) 0.087	(–) 0.304	(+) 0.142	(+) 0.511	(+)0.760	(+)0.053	(+)0.445	(–)0.620	(–) 0.174	(–) 0.638	(−) 0.199	(−) 0.378
15–5	(–) 0.737	(+) 0.928	(–) 0.402	(–) 0.582	(+)0.305	(–)0.545	(+)0.001	(+)0.140	(+) 0.195	(+) 0.599	(+) 0.036	(+) 0.404

* Significant correlation (*p < *0.05): MW (molecular weight); very strong (0.76–0.99); strong (0.51–0.75); fair (0.26–0.50); weak (0).

## Conclusion

Analyzing protein MW through SDS-PAGE provides a promising approach for assessing semen quality in indigenous Indonesian buffalo bulls. Although the semen quality of the buffaloes in this study was variable, all bulls met the established Indonesian standards for semen quality and exhibited adequate fertilization potential. These results provide valuable insights into the reproductive biology of Indonesian buffalo bulls and form the basis for predicting fertility capacity through a comprehensive analysis of sperm characteristics and molecular profiles of sperm proteins.

## List of abbreviations

AI, artificial insemination; AIC, artificial insesmination center; AR, acrosome reaction; ALH, amplitude lateral head displacement; BCA, bicinchoninic acid; BCF, beat cross frequency; ATP, adenosine triphosphate; CASA, computer-assisted semen analysis; CMA3, chromomycin A3; DNA, deoxyribonucleic acid; kDa, kilodalton; MW, molecular weight; pMOT, progressive motility; SDS-PAGE, sodium dodecyl sulfate-polyacrylamide gel electrophoresis; STR, straightness; VAP, velocity of the average path; VCL, curvilinear velocity; VSL, velocity straight line; WOB, wobble.

## References

[1] Maulana T, Iskandar H, Said S, Gunawan A (2023). The current status and potential development of genetic resources of indigenous Toraya spotted buffalo in Indonesia: a systematic review. World Vet J.

[2] Vargas-Ramella M, Pateiro M, Maggiolino A, Faccia M, Franco D (2021). EBuffalo milk as a source of probiotic functional products. Microorganisms.

[3] Tanga BM, Qamar AY, Raza S, Bang S, Fang X, Yoon K (2021). Semen evaluation: methodological advancements in sperm quality-specific fertility assessment-a review. Anim Biosci.

[4] Fernandez-Novo A, Santos-Lopez S, Barrajon-Masa C, Mozas P, de Mercado E, Caceres E (2021). Effect of extender, storage time and temperature on kinetic parameters (CASA) on bull semen samples. Biology.

[5] Prabowo TA, Bintara S, Yusiati LM, Sitaresmi PI, Widayati DT (2023). Evaluation deoxyribonucleic acid (DNA) fragmentation of local Indonesian cattle frozen sperm using Halomax^˙^ method. Biodiversitas.

[6] Gebreyesus G, Lund MS, Kupisiewicz K, Su G (2021). Genetic parameters of semen quality traits and genetic correlations with service sire nonreturn rate in Nordic Holstein bulls. J Dairy Sci.

[7] Dcunha R, Hussein RS, Ananda H, Kumari S, Adiga SK, Kannan N (2022). Current insights and latest updates in sperm motility and associated applications in assisted reproduction. Reprod Sci.

[8] Gallo A, Esposito MC, Tosti E, Boni R (2021). Sperm motility, oxidative status, and mitochondrial activity: exploring correlation in different species. Antioxidants (Basel).

[9] Bucci D, Spinaci M, Bustamante-Filho IC, Nesci S (2022). The sperm mitochondria: clues and challenges. Anim Reprod.

[10] Cho MJ, Kim YJ, Yu WD, Kim YS, Lee JH (2021). Microtubule integrity is associated with the functional activity of mitochondria in HEK293. Cells.

[11] Ansari MS, Rakha BA, Akhter S (2017). Cryopreservation of nili-ravi buffalo (*Bubalus bubalis*) semen in AndroMed^®^ extender; *in vitro* and *in vivo* evaluation. Reprod Domest Anim.

[12] Chenoweth PJ (2005). Genetic sperm defects. Theriogenology.

[13] Sun W, Jiang S, Su J, Zhang J, Bao X, Ding R (2020). The effects of cryopreservation on the acrosome structure, enzyme activity, motility, and fertility of bovine, ovine, and goat sperm. Anim Reprod.

[14] Iskandar H, Sonjaya H, Arifiantini RI, Hasbi H (2022). Correlation between semen quality, libido, and testosterone concentration in Bali bulls. JITV.

[15] Rajabi-Toustani R, Akter QS, Alamadaly EA, Hoshino Y, Adachi H, Mukoujima K (2019). Methodo-logical improvement of fluorescein isothiocyanate peanut ag- glutinin (FITC-PNA) acrosomal integrity staining for frozen-thawed Japanese Black bull spermatozoa. J Vet Med Sci.

[16] Hasbi H, Dagong MIA, Zulkharnain Z, Baba S, Sonjaya H, Baco S (2023). Comparison of fresh and cryopreserved semen quality of polled and horned bali bulls. Irani J Appl Anin Sci.

[17] Baharun A, Setiawan AB, Rahmia A, Iskandar H, Gunawan M, Anwar S (2023). Frozen semen characteristics of limousin bull at different ages. Trop Anim Sci J.

[18] Carreira JT, Trevizan JT, Carvalho IR, Kipper B, Rodrigues LH, Silva C (2017). Does sperm quality and DNA integrity differ in cryopreserved semen samples from a young, adult, and aged Nellore bulls?. Basic Clin Androl.

[19] Kumaresan A, Gupta MD, Datta TK, Morrell JM (2020). Sperm DNA integrity and male fertility in farm animals: a review. Front Vet Sci.

[20] D’Occhio J, Hengstberger KJ, Tutt D, Holroyd RG, Fordyce G, Boe-Hansen GB (2013). Sperm chromatin in beef bulls in tropical environments. Theriogenology.

[21] Ribas-Maynou J, Benet J (2019). Single and double strand sperm DNA damage: different reproductive effects on male fertility. Genes.

[22] Iskandar H, Sonjaya H, Arifiantini RI, Hasbi H (2022). The quality of fresh semen and frozen semen and its correlation with molecular weight of seminal plasma protein in Bali cattle. Trop Anim Sci J.

[23] Fernández-López P, Garriga J, Casas I, Yeste M, Bartumeus F (2022). Predicting fertility from sperm motility landscapes. Commun Biol.

[24] Kumar D, Kumar P, Singh P, Yadav SP, Yadav PS (2016). Assessment of sperm damages during different stages of cryopreservation in water buffalo by fluorescent probes. Cytotechnology.

[25] Inanc ME, Beste C, Tekin K, Alemdar H, Daskin A (2018). The combination of CASA kinetic parameters and fluorescein staining as a fertility tool in cryopreserved bull semen. Turkish J Vet Anim Sci.

[26] Jia B, Liang J, Lv C, Memon S, Fang Y, Wu G (2021). The characteristics of proteome and metabolome associated with contrasting sperm motility in goat seminal plasma. Sci Rep.

[27] Guasti PN, Souza FF, Scott C, Papa PM, Camargo LS, Schmith RA (2020). Equine seminal plasma and sperm membrane: functional proteomic assessment. Theriogenology.

[28] Fu Q, Pan L, Huang D, Wang Z, Hou Z, Zhang M (2019). Proteomic profiles of buffalo spermatozoa and seminal plasma. Theriogenology.

[29] Iskandar H, Andersson G, Sonjaya H, Arifiantini RI, Said S, Hasbi H (2023). Protein identification of seminal plasma in Bali bull (*Bos**javanicus*). Animals.

[30] Moura AA, Souza CE, Stanley BA, Chapman DA, Killian GJ (2010). Proteomics of cauda epididymal fluid from mature Holstein bulls. J Proteomics.

[31] Nixon B, Bromfield EG, Dun MD, Redgrove KA, McLaughlin EA, Aitken RJ (2015). The role of the molecular chaperone heat shock protein A2 (HSPA2) in regulating human sperm-egg recognition. Asian J Androl.

[32] Chen T, Lin T, Li H, Lu T, Li J, Huang W (2018). Heat shock protein 40 (HSP40) in Pasific white shrimp (*Litopenaeus vannamei*): Molecular cloning, tissue distribution and ontogeny, response to temperature, acidity/alkalinity and salinity stresses, and potential role in ovarian development. Front Physiol.

[33] Rosyada ZNA, Ulum MF, Tumbelaka LITA, Solihin DD, Purwantara B, Memili E (2022). Implications of sperm heat shock protein 70-2 in bull fertility. Vet World.

[34] Fukuda M, Sakase M, Fukushima M (2016). Changes of IZUMO1 in bull spermatozoa during the maturation, acrosome reaction, and cryopreservation. Theriogenology.

[35] Kim E, Kim JS, Lee Y, Song BISA, Sim BW, Kim SU (2013). Molecular cloning, characterization of porcine IZUMO1, an IgSF family member. Reprod Domest Anim.

[36] Kausar R, Hameed A, Jamil H, Iqbal Z, Bahadur SUK, Jabbar J (2023). Comparative analysis of buffalo and cow milk for quality characteristics and ß-N-acetyl-glucosaminidase activity in non-infected animals. Emir J Food Agric.

[37] Pei J, Pan X, Wei G, Hua Y (2023). Research progress of glutathione peroxidase family (GPX) in redoxidation. Front Pharmacol.

[38] Rosyada ZNA, Ulum MF, Tumbelaka LITA, Purwantara B (2020). Sperm protein markers for Holstein bull fertility bull fertility at National Artificial Insemination Centers in Indonesia. Vet World.

[39] Rodríguez-Villamil P, Hoyos-Marulanda V, Martins JAM, Oliveira AN, Aguiar LH, Moreno FB (2016). Purification of binder of sperm protein 1 (BSP1) and its effects on bovine *in vitro* embryo development after fertilization with ejaculated and epididymal sperm. Theriogenology.

[40] Nagy S, Johannisson A, Wahlsten T, Ijäs R, Andersson M, Rodriguez-Martinez H (2013). Sperm chromatin structure and sperm morphology: their association with fertility in AI-dairy Ayrshire sires. Theriogenology.

